# Endothelial *TIE2* Mutation Induced Contraction Deficiency of Vascular Smooth Muscle Cells via Phenotypic Transition Regulation in Venous Malformations

**DOI:** 10.7150/ijms.102700

**Published:** 2025-05-07

**Authors:** Zhong Du, Fan Yu, Yuan He You, Zhi Yang Zhao, Zhuo Wei Tian, Meng Xiao, Yan An Wang

**Affiliations:** Department of Oral and Maxillofacial-Head and Neck Oncology, Shanghai Ninth People's Hospital, Shanghai Jiao Tong University School of Medicine; College of Stomatology, Shanghai Jiao Tong University; National Center for Stomatology; National Clinical Research Center for Oral Diseases; Shanghai Key Laboratory of Stomatology; Shanghai Research Institute of Stomatology; Shanghai Center of Head and Neck Oncology Clinical and Translational Science, Shanghai 200011, China.

**Keywords:** Venous malformations, Mutation, Endothelial cell, TIE2, Mouse model, Phenotypic transition, Vascular smooth muscle cell.

## Abstract

**Introduction:** Venous malformations (VMs) are congenital vascular malformations characterized by venous cavity enlargement and malformation. Although *TIE2* mutation is a recognized genetic landscape in VMs, the regulatory role of *TIE2* in vascular smooth muscle cell (VSMC) contraction remains unclear.

**Materials and Methods:** We generated *Tie2-R848W^fl/fl^;Tie2^Cre+^* and* Tie2-R848W^fl/fl^;Apln^ER+^* mice through specific expression of *Tie2-R848W*, a typical mutation in inherited VM, in endothelial cells (ECs). Histological and transcriptome sequencing analyses were performed on vascular abnormalities in the mutant mouse model. Postnatal vascular development *in vivo* was studied through morphometric analysis of the retinal vasculature. Under *in vitro* coculture conditions, the functional abnormality of VSMCs was studied using transwell analysis, proliferation analysis, a cell contraction assay and transcriptome sequencing analysis. Markers related to the VSMC phenotypic transition were analyzed via western blotting and quantitative RT‑PCR.

**Results:** *Tie2-R848W^fl/fl^;Tie2^Cre+^* mice developed spontaneous pulmonary vascular malformations displaying internal hemorrhage and increased vasculature with α-SMA+ enveloped VSMCs. In* Tie2-R848W^fl/fl^;Apln^ER+^* mice,* Tie2-R848W* mutation also induced postnatal retinal vascular malformations (higher vascular density and coverage of α-SMA+ VSMCs). According to phenotypes and molecular markers (Acta2, Cnn1, Sm22a and Opn), dysregulated phenotypic transition of VSMCs might be the pathogenic basis. Under *in vitro* coculture condition, the decreased contractile ability of synthetic VSMCs was significant in the mutant group, while downregulated ion transmembrane transport and TNFSF10 may play substantial roles in initiating this process.

**Conclusion**: Endothelial *TIE2* mutation might induce an abnormal EC-VSMC regulatory relationship strongly associated with phenotypic transition of VSMCs. Weakened contractility and abnormal proliferation induce chronic cavity expansion and thickening of the muscle layer, which may be potential mechanism basis of VMs.

## Introduction

Venous malformations (VMs) are congenital vascular malformations characterized by chronically enlarged and malformed venous cavities. VMs can be present in diverse tissues or organs and induce various painful symptoms, such as swelling, bleeding, localized intravascular coagulopathy, pulmonary embolism and deformity. Based on congenital characteristics, somatic or inherited variants have been identified in VM lesions^1^. According to novel classification suggestions from the International Society for the Study of Vascular Anomalies (ISSVA), vascular endothelial tyrosine kinase receptor (*TIE2*) and *PIK3CA* are commonly recognized as the most frequently carried mutated genes associated with diverse types of VMs. Based on a systematic review of genetic studies on human VMs and our reported verification^2^, ^3^, compared with *PIK3CA* mutations, *TIE2* mutations are more typical and frequent genetic findings in common VMs, especially those of the head and neck region. Among the diverse *TIE2* mutation sites and types reported, *TIE2-R849W* was the first identified mutation and is the most common mutation in the only type of autosomal recessive VM, multiple cutaneous and mucosal VM (VMCM)^3^-^5^. Thus, a deep and systematic investigation into this particular mutation is critical for elucidating the mechanisms underlying *TIE2* mutation-related VMs.

Constructing an animal genetic model to identify and analyze the driving role of *TIE2* mutation in VM formation is a challenging but necessary process. Unfortunately, aside from human umbilical vein endothelial cell (HUVEC) line-derived or patient-derived xenograft nude murine models^6^, ^7^, the transgenic zebrafish model constructed in our previous work is the only *TIE2*-related genetically modified animal model for VMs^8^. Furthermore, angiogenesis is a complex and highly regulated process, and the embryonic stage cannot be fully mimicked in a nude mouse xenograft model. Inspired by a transgenic mouse model with a VM-related *PIK3CA* mutation^9^, ^10^, we constructed a genetically modified knock-in mouse model to focus on the classical inheritable *TIE2-R849W* mutation. Based on the precise spatiotemporal regulation of the expression pattern, we analyzed the angiogenic process and other phenotypes to explore the pathogenic mechanism of *TIE2* mutation in VMs. According to previous studies on VMs, in addition to abnormal activation of the inner signaling network of endothelial cells (ECs)^3^, ^11^, a dysregulated relationship between ECs and vascular smooth muscle cells (VSMCs) might play a vital role in the formation and development of VMs. Most related studies have focused on the recruitment deficiency of VSMCs under the influence of *TIE2* mutation in ECs^7^, ^12^. However, few investigations have been performed to examine alterations in the contractile function and strength of VSMCs, which may also play important roles in VM formation^13^.

In this study, we observed changes in vascular phenotype in a novel genetically modified mouse model and performed histological examination and transcriptome sequencing analysis. Retinal vascular development was also quantitatively analyzed, as this process is a common and important model of early vascular development *in vivo*. *TIE2-R849W*-related HUVEC model was also constructed via lentivirus transfection to verify the effect of this mutation on the functions of VSMCs. By comparing the transcriptomic expression data *in vivo* and *in vitro*, we determined the possible mechanism of *TIE2-R849W*-related VMs.

## Materials and Methods

### Mouse models and husbandry

All animal experiments were conducted in accordance with guidelines from the US National Institutes of Health (NIH Publication No. 85-23, revised 1996), and were approved by the Animal Care and Use Committee of Shanghai Ninth People's Hospital. Via homologous comparison of amino acid sequence and genetic sequence, site R849 of homo sapiens TIE2 corresponds to site R848 of mus musculus TIE2 (Figure S1A). Then, human VMCM related mutation *TIE2-R849W* corresponds to *Tie2-R848W* in mus musculus gene. Based on cDNA sequence information and transcription rules, the genetic site change (3' to 5') *CGG*>*TGG* was confirmed as a core insert element for genome editing (Figure S1B). All F1 generation mice accepted Sanger Sequencing to confirm target genetic variant (Figure S2), *CGG*>*TGG*. Genotypes were determined by tail-snip PCR amplification.

*Apln^ER^* mice were constructed and kindly provided by Prof. Bin Zhou, Shanghai Institute of Biochemistry and Cell Biology, Chinese Academy of Science (Shanghai, China)^14^.* H11^mGmT^*, *Tie2-R848W^fl/fl^* and *Tie2^Cre^* mice on C57BL/6J were constructed and provided by Gempharmatech Co., Ltd, the Model Animal Research Center (MARC) of Nanjing University (Nanjing, China). All mice were housed in specific pathogen-free and temperature-controlled facility accredited by Association for Assessment and Accreditation of Laboratory Animal Care International (AAALAC). *Tie2^Cre+^* mice were crossed with *H11^mGmT^* to obtain *Tie2^Cre+^;H11^mGmT^* mice. *Apln^ER+^* mice were crossed with *H11^mGmT^* to obtain *Apln^ER+^;H11^mGmT^* mice. *Tie2^Cre+^* or *Tie2^Cre-^* mice were crossed with *Tie2-R848W^fl/fl^* to obtain *Tie2-R848W^fl/fl^;Tie2^Cre+^* or *Tie2-R848W^fl/fl^;Tie2^Cre-^* mice. *Apln^ER+^* or *Apln^ER-^* mice were crossed with *Tie2-R848W^fl/fl^* to obtain *Tie2-R848W^fl/fl^; Apln^ER+^* or *Tie2-R848W^fl/fl^; Apln^ER-^* mice. For *Apln^ER^* mice, to activate Cre-mediated recombination, tamoxifen was dissolved in corn oil and injected intraperitoneally according to prepense designed time. All mice were euthanized by cervical dislocation or carbon dioxide asphyxiation. The ethical principles established by the National Institutes of Health Guide for the Care and Use of Laboratory Animals (NIH Publications No. 8523, revised 2011) were followed. This animal study had been approved by the Ethics Committee of Shanghai Ninth People's Hospital (HKDL[2018]39).

### Immuno-staining analysis

All tissue was fixed, dehydrated, embedded in paraffin, sectioned at 3 μm and stained with hematoxylin and eosin according to standard protocols. For common immunohistochemistry staining, primary antibody incubation was performed with anti-CD34 (1:50, 14486-1-AP, Proteintech, USA), anti-SMA (1:100, 55135-1-AP, Proteintech, USA), anti-ERG (1:1200, 14356-1-AP, Proteintech, USA), anti-VEGFA (1:100, 55135-1-AP, Proteintech, USA) and anti-TIE2 (1:50, ab218051, Abcam, USA). The sections were incubated with horseradish peroxidase (HRP)-labeled secondary antibodies (GK500705, Gene Tech, China). For immunofluorescence staining, primary antibody incubation with anti-CD31 (1:100, 553370, Ms CD31 Pure MEC 13.3, BD, USA), anti-SMA (1:100, 48938, CST, USA) was performed and incubated overnight at 4 ℃. The retinas/sections were washed and incubated with secondary antibodies^9^, including anti-rabbit antibody (1:200, 712-225-150, Cy™2 AffiniPure Donkey Anti-Rat, Jackson, USA) and anti-mouse antibody (1:200, 715-165-150, Cy™3 AffiniPure Donkey Anti-Mouse, Jackson, USA), overnight at 4 ℃, and nucleus was stained with DAPI (1:200, 4083, CST, USA). Images were finally scanned by an inverted fluorescence microscope (Nikon, Tokyo, Japan, 10/20 ×) and photographed at 492 nm and 550 nm.

### Morphometrical analysis of retinal vascular

Newborn pups (postnatal 2 days, P2) were injected intragastrically with tamoxifen (5 mg/ml.). Retinal angiogenesis was studied at P7 and P15. For each experiment, mice from 3 litters were used to account for intrauterine and interindividual variability. The eyeballs were removed and quickly placed on ice. PBS solution (2X) was pre-added to fix eyeballs for 10 min. The lens and vitreous body were then removed. 4 radial incisions reaching about 2/3 of the radius of the retina were made to form a "petal" shape. The retina was fixed in 4% paraformaldehyde at -20℃ for at least 20min. Retina was washed by PBST, and was fixed by FBS at room temperature for 1 hour. AngioTool software (Version 0.6a, NCBI, USA) was used to model the fluorescence images of retinal vessels and analyze the vascular radial expansion, coverage ratio of VSMC, vascular density, and number of end points. GraphPad Prism (Version 8, GraphPad Software, USA) was used for plotting and statistical analysis of relevant data.

### RNA extraction, sequencing, and data Analysis

The cells/tissue were harvested and sent to OE biotech Co., Ltd (Shanghai, China) for RNA sequencing. RNA purity was evaluated using the NanoDrop 2000 spectrophotometer (Thermo Scientific, USA). The libraries were sequenced on an Illumina HiSeq X Ten platform, and 150 bp paired-end reads were generated. FPKM of each gene was calculated using Cufflinks, and the read counts of each gene were obtained by HTSeq-count. The published microarray data related to mutant HUVECs was acquired from National Center for Biotechnology in the Information (NCBI) Gene Expression Omnibus and are available through GEO series accession number GSE46684^15^. The differentially expressed gene analysis was performed using the DESeq (2012) R package. The *p* value < 0.05 and the fold change > 2 were set as the threshold for significantly differential expression of genes.

### Cell Culture

Human umbilical vein endothelial cells (HUVECs, Catalog#8000, 5×10^5^ cells in 1 ml volume at passage one and delivered frozen) and human umbilical vein smooth muscle cells (HUVSMCs, Catalog#8020, 5×10^5^ cells in 1 ml volume at passage one and delivered frozen) were all purchased from ScienCell Research Laboratories (USA). As suggested, HUVECs were cultured in the Endothelial Cell Medium (ECM, 1001, ScienCell, USA), while HUVSMCs were cultured in the Smooth Muscle Cell Medium (SMCM, 1101, ScienCell, USA), in an incubator with 37 °C and 5% CO_2_ atmosphere. Plasmids and lentiviruses were synthesized by RiboBio Inc. (China). Cell transfection was performed using the Lipofectamine®3000 Transfection Kit (Invitrogen, USA). Before co-culturing, HUVECs and HUVSMCs were cultured 1 day in advance with medium starvation (without serum or growth factor). On the next day, 3 μm sterile Transwell co-culture cells were placed in 6-well plates. HUVEC was inoculated in the lower chamber with 10 × 10^4^ cells/well (without serum or growth factor), and HUVSMC was inoculated with 30×10^4^ cells/well in the upper chamber (without serum or growth factor).

### Transwell analysis

Transwell chamber (8 μm, Millipore, BD, USA) was placed in a 24-well plate. The lower chamber of the plate was inoculated with HUVEC at a rate of 14×10^4^ cells/well (without serum or growth factor), and the upper chamber was inoculated with HUVSMC at a rate of 42×10^4^ cells/well (without serum or growth factor). Whole chambers were cultured in an incubator with 37 °C and 5% CO_2_ atmosphere for 24 hours. Invaded cells were stained with 0.1% crystal violet liquid. The number of invaded cells were counted using ImageJ (Version 1.8.0, Rawak, Germany) after taking photos.

### Cellular proliferation analysis

HUVECs/HUVSMCs were inoculated into 96-well plates with 1,500 cells/well under different treatments. Subsequently, Cell Counting Kit-8 liquid (CK04, Dojindo, Japan) was directly added to each well according to prepense designed time respectively, and then transferred to an incubator at 37 °C for 2 hours to measure the absorbance at 450 nm.

### Western blot analysis

Total protein was isolated from whole-cell lysates using lysis buffer with protein phosphatase inhibitor cocktail (New Cell & Molecular Bitotec, China). After the lysates were subjected to SDS-PAGE electrophoresis, proteins were transferred by electroblotting. The membranes were then blocked and incubated with primary antibodies against TIE2 (1:1000, ab218051, Abcam, USA), pTIE2 (1:1000, 4221S, CST, USA), SM22A (1:1000, 10493-1-AP, Proteintech, USA), SMA (1:1000, 55135-1-AP, Proteintech, USA), OPN (1:1000, 22952-1-AP, Proteintech, USA), TNFSF10 (1:1000, 27064-1-AP, Proteintech, USA), FOXO1(1:1000, 2880, CST, USA), pFOXO1(1:1000, 9461, CST, USA), ACTIN (1:1000, 3700, CST, USA), GAPDH (1:1000, 5174, CST, USA). After incubating with peroxidase-linked secondary antibodies (Cell Signaling Technology, USA), the membranes were washed and visualized using Immobilon Western HRP Chemiluminescence Substrate (Millipore, USA).

### Quantitative RT‑PCR

PrimeScript RT reagent Kit (Takara, Japan) was used to extract total RNA, which were reversely transcribed next following the manufacturer's protocol. Then, the cDNA was subjected to qRT-PCR detection for *ACTA2*, *CNN1*, *TAGLN*, *OPN*, *KCNA1*, *KCNMA1*, *KCNB2*, *KCNJ5*, *KCNJ6*, *KCNJ9*, *KCNQ5*, *GAPDH* using a SYBR Green Premix Kit (Takara, Japan). The validation of the RT-PCR was verified by three independent experiments. For each independent experiment, three repeated holes were set in each group. The relative expression was calculated using the 2-ΔΔCT method. The PCR primers were described in Table S1.

### Cell contraction assay

Cell contraction assay was performed via Cell Contraction Assay Kit (CBA-201, Cell Biolabs, USA)^16^. Cells were harvested and resuspended at 5×10^6^ cells/ml. Prepare the collagen lattice by mixing 2 parts of cell suspension and 8 parts of cold Collagen Gel Working Solution. Cell-collagen mixture was then added and incubated 1 hour at 37ºC. After collagen polymerization, culture medium was added atop each collagen gel lattice and incubated for 2 days. After gently releasing collagen gels from the sides of the culture dishes with a sterile spatula, the collagen gel size change could be measured at designed times with a ruler.

### Statistical Analysis

GraphPad Prism (Version 8, GraphPad Software, USA) was applied for statistical analyses. Student's t-test was used to determine the significance of difference between two groups. It was considered statistically significant when *p* values < 0.05. The data were presented as the mean ± SD.

## Results

### *Tie2-R848W^fl/fl^;Tie2^Cre+^* mice developed spontaneous pulmonary vascular deficiency displaying internal hemorrhage and increased quantity of vasculature with obvious α-SMA+ enveloped VSMCs

**Figure 1:**
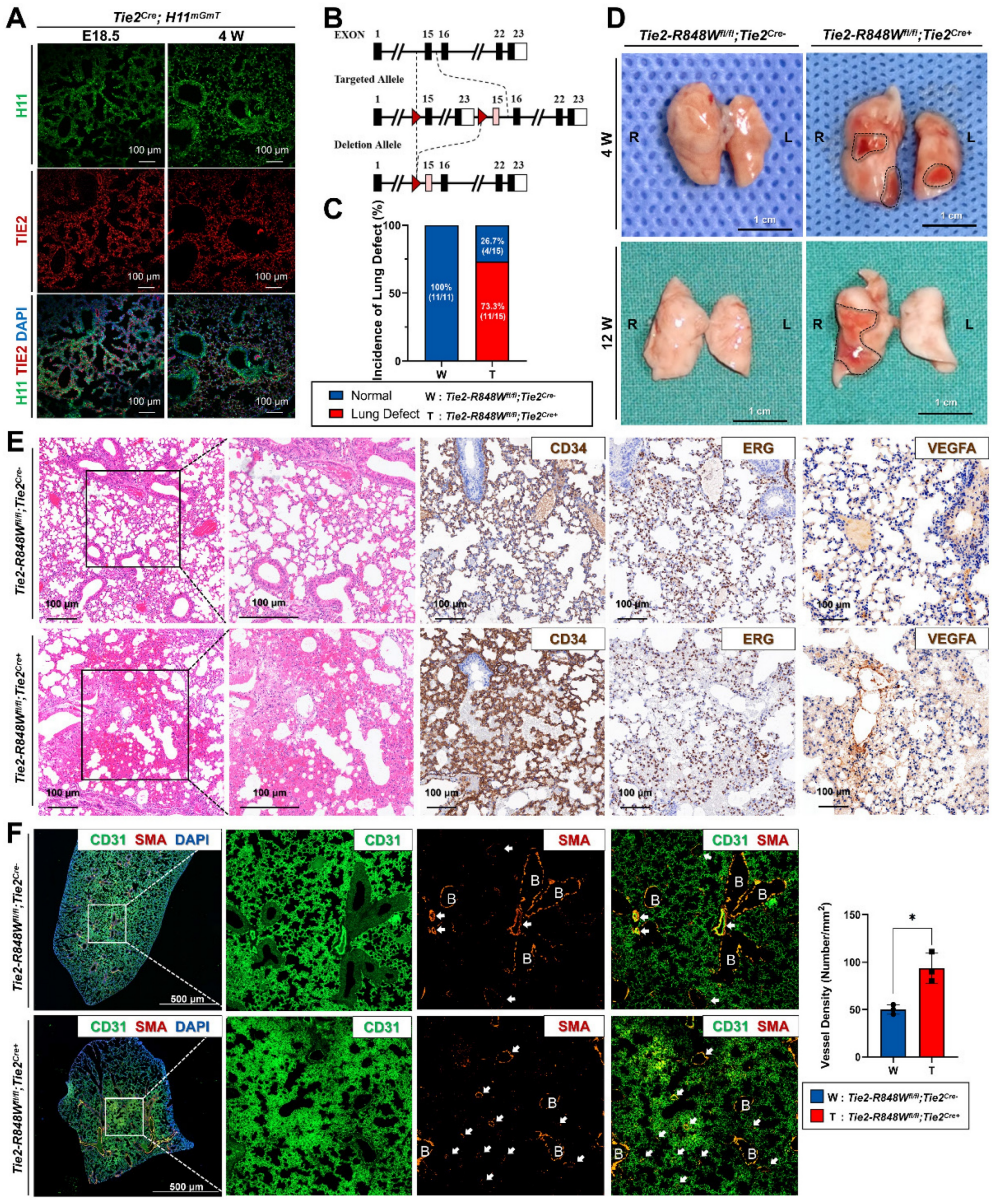
***Tie2-R848W^fl/fl^;Tie2^Cre+^* mice developed pulmonary vascular malformations with internal hemorrhage. (A)** High Cre-inducible efficiency (red) in the lungs was apparent for either the embryonic period (E18.5) or the immature period (W4). Bar=100 μm. **(B)** Via the CRISPR/Cas9 genetic editing approach and based on a Cre/loxP recombination design, an inducible knock-in mouse model, *Tie2-R848W^fl/fl^* transgenic mice, was generated. WT (top), targeted inducible *Tie2-R848W* locus (middle), and Cre-loxP-mediated deletion of exons 15-23 encoding the wild-type domain of *Tie2* (bottom). Red triangles, *loxP* sites. Pink rectangle, mutant exon 15. **(C)** Compared to no mice in the control group (0/11), 73.3% (11/15) of *Tie2-R848W^fl/fl^;Tie2^Cre+^* mice exhibited regional red spots in the lung lobes. **(D)** Regional red spots (surrounded by black dotted line) in the lung lobes could be observed at W4 and W12. Bar=1 cm. **(E)** Immunohistochemical analysis for lung defects (CD34, ERG and VEGFA) confirmed numerous internal hemorrhages in a no-boundary region without obvious lymphatic cell infiltration, the distribution of vascular ECs, and an enlarged alveolar space filled with erythrocytes. Bar=100 μm. **(F)** In the hemorrhage region, a greater amount of vasculature (indicated by white arrow) with discontinuous α-SMA+ enveloped VSMCs was detected via immunofluorescence colocalization analysis for CD31 (green) and α-SMA (orange). B, bronchus. Bar=500 μm. The vascular density (vascular number/mm^2^) was quantified in lung tissues by analyzing equivalent regions from both the control and mutant groups. *P<0.05.

According to the positive red fluorescence indicating *Tie2^Cre+^;H11^mGmT^*, Tie2 was present in the lungs, heart, spleen, kidneys, tongue and other tissues with rich vasculature (embryonic day [E] 18.5 and week [W] 4) (Figure S1 and S2). Among these organs, the lungs exhibited the most apparent high Cre inducible efficiency in both the embryonic period (E18.5) and the immature period (W4) (Figure 1A). Via a CRISPR/Cas9 genetic editing approach and fluorescence analysis of Cre inducible efficiency and based on the Cre/loxP recombination design, we generated an inducible knock-in mouse model, *Tie2-R848W^fl/fl^* transgenic mice (Figure 1B and Figure S1-4).

Endothelial-specific mutant mice were obtained by crossing *Tie2-Cre* mice and *Tie2-R848W^fl/fl^* mice. Similar to the finding that human VMs occur more frequently in female patients than in male patients^2^, the female proportion of homozygous mutant mice (66.7%, 10/15) was also slightly but nonsignificantly higher than that of male mice (Figure S5). Based on anatomical examination and observation of the skin, oral mucosa and internal organs (lungs, heart, liver, kidneys, spleen and other organs that are histologically rich in vascular tissue) at W4 and W12 (Figure S6), compared to the control mice (0/11), 73.3% (11/15) of *Tie2-R848W^fl/fl^;Tie2^Cre+^* mice exhibited regional red spots in the lung lobes; however, no obvious abnormalities were found in other organs (Figure 1C and D). There was no obvious pattern for the distribution of lesions in the lung lobes. No apparent reduction in ordinary activity, breathing difficulty or death was observed in the mutant mouse group.

Compared to normal lung lobe of the control group, histological analysis of the regional red spots from *Tie2-R848W^fl/fl^;Tie2^Cre+^* mice (W4) was performed, which indicated numerous internal hemorrhages in a no-boundary region in *Tie2-R848W^fl/fl^;Tie2^Cre+^* mice compared with the normal lung lobes of the controls (Figure 1E). Immunohistochemical analysis (of CD34, ERG and VEGFA) confirmed the distribution of vascular ECs and the enlargement of the alveolar space filled with erythrocytes. The expression of TIE2 was also confirmed to be consistent with CD34 levels in mutant mice (Figure S7). Immunofluorescence analysis of colocalization (CD31 and α-SMA) in the hemorrhaged region revealed an increased quantity of vasculature with obvious α-SMA+ enveloped VSMCs (Figure 1F), which might have been a key reason for the hemorrhage. In the normal lung lobes of the control group, obvious α-SMA+ enveloped VSMCs were detected only around the relatively few scattered veins or arteries. To further verify the somatic *TIE2* mutation pattern in the transgenic mutant mouse model, considering the similar positive expression efficiency in pulmonary ECs (Figure S4 and S8), tamoxifen was intraperitoneally administered to the *Tie2-R848W^fl/fl^;Apln^ER+^* mice at W4. Similar but slight pulmonary vascular malformations with hemorrhaging were also identified in *Tie2-R848W^fl/fl^;Apln^ER+^ mice* at W12 (Figure S8B).

The above information indicated the potential association between the changes in pulmonary vascular malformations with internal hemorrhage in mutant mice and the activation of mutant genes.

### The EC-specific *Tie2-R848W* mutation induced postnatal retinal vascular malformations

**Figure 2:**
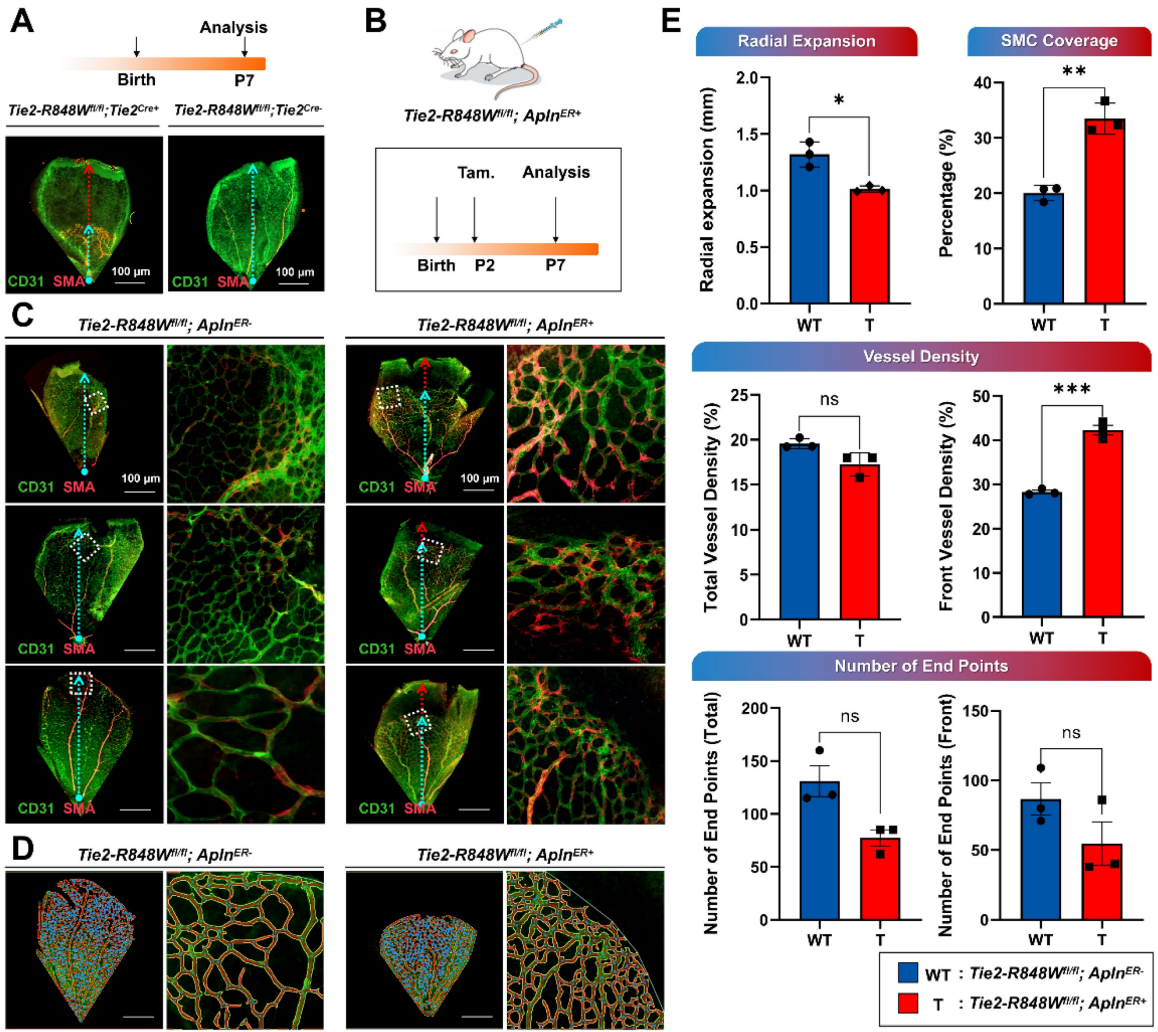
**Endothelial cell-specific* Tie2-R848W* induced postnatal retinal vascular malformations. (A)** Reduced retinal vascular outgrowth was identified in *Tie2-R848W^fl/fl^;Tie2^Cre+^* mice at P7 via immunofluorescence colocalization analysis for CD31 (green) and α-SMA (orange). Blue dashed arrow, radial expansion of the retinal vasculature from the outer front edge of the vascular network to the center of the optic axis. Red dashed arrow, distance between the retinal vasculature margin and retinal margin (reduced retinal vascular outgrowth). Bar=100 μm. **(B)** Schematic of tamoxifen (Tam.) administration and analysis in the *Tie2-R848W^fl/fl^;Apln^ER+^* mice for postnatal retinal vascular analysis (P7). **(C)** Immunofluorescence colocalization analysis of CD31 (green) and α-SMA (orange) in the postnatal retinal vasculature (P7) in *Tie2-R848W^fl/fl^;Apln^ER+^* mice. Blue dashed arrow, radial expansion of the retinal vasculature from the outer front edge of the vascular network to the center of the optic axis. Red dashed arrow, distance between the retinal vasculature margin and retinal margin (reduced retinal vascular outgrowth). Square region surrounded by white dotted line, typical retinal vascular growth front region. Bar=100 μm. **(D)** Microvascular plexuses at retinal locations spanning the whole region and growth front region were modeled digitally in *Tie2-R848W^fl/fl^;Apln^ER+^* mice with AngioTool software. Blue spot, junction point of vascular branches. Yellow line, outline of the vasculature. Red line, skeleton and direction of the vasculature. Bar=100 μm. **(E)** According to modeling analysis, in the *Tie2-R848W^fl/fl^;Apln^ER+^* mouse group, the radial expansion distance was significantly decreased, while the coverage rate of α-SMA+ VSMCs was significantly increased. Although total vascular density was not significantly different from that in the control group, the vascular density of the developmental frontier area in mutant mice was significantly increased. Although the number of neovascular buds (the number of end points) was slightly decreased in the mutant group, no significant difference was found. *P<0.05, **P<0.001, ***P<0.0001, ns, not significant.

Reduced retinal vascular outgrowth was identified in *Tie2-R848W^fl/fl^;Tie2^Cre+^* mice at postnatal day 7 (P7), consistent with the use of this model a classical postnatal retinal vascular model (Figure 2A). *Tie2-R848W^fl/fl^;Apln^ER+^* mice were further evaluated to precisely characterize the function of the *Tie2-R848W* mutation in the postnatal vasculature. Tamoxifen was administered at P2 to activate Cre-mediated mutation in the ECs (Figure 2B). P7 retinas were utilized to assess vascular development under the influence of *Tie2* mutation. Immunofluorescence colocalization analysis (for CD31 and α-SMA) revealed obviously reduced retinal vascular outgrowths enveloping more discontinuous α-SMA+ VSMCs in *Tie2-R848W^fl/fl^;Apln^ER+^* mice (Figure 2C). The images extracted from the different microvascular plexuses at retinal locations spanning the whole region and the front growth region were analyzed using AngioTool software (Figure 2D).

Statistical analysis revealed that retinal vascular formation and elongation were significantly downregulated in the mutant group from the outer front edge of the vascular network to the center of the optic axis (radial expansion distance) (Figure 2E; *Tie2-R848W^fl/fl^;Apln^ER+^* mice at P7). The coverage rate of α-SMA+ VSMCs in the mutant group was significantly higher than that in the control group, indicating that the proliferation ability of smooth muscle was significantly upregulated, which implies a phenotypic transition (Figure 2E). The vascular density was calculated in the total and developmental frontier areas of the retinal vascular network and found to be significantly increased in mutant mice, similar to the phenotype of pulmonary vascular malformation (Figure 2E). Although the number of neovascular buds (end points) was slightly decreased in the mutant group, no significant difference was found (Figure 2E). To evaluate the long-term vascular reconstruction process, retinal vascular development in *Tie2-R848W^fl/fl^;Apln^ER+^* mice was assessed again at P15. No obvious difference was observed, implying that *Tie2-R848W* might significantly influence early angiogenesis but not vascular reconstruction (Figure S9).

In conclusion, EC-specific activation of the *Tie2-R848W* mutation induced postnatal retinal vascular malformations. In addition, the pulmonary and retinal phenotypes in mutant mice showed similarities in different aspects (higher vascular density and higher coverage rate of α-SMA+ VSMCs).

### Dysregulated function and phenotypic transition of vascular smooth muscle might be the pathogenic basis of *Tie2* mutation-related vascular malformations

**Figure 3:**
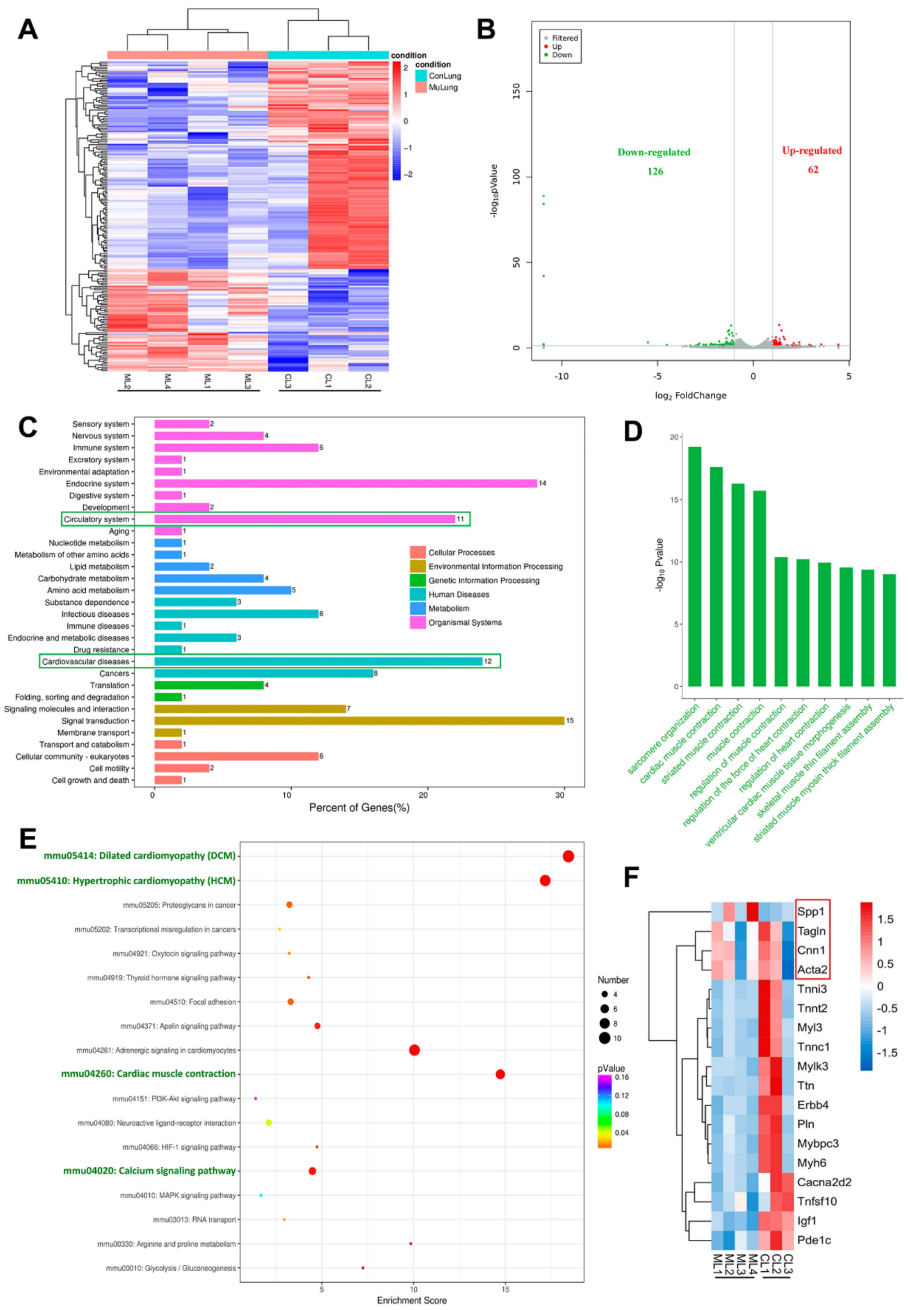
** Transcriptomic analyses of pulmonary vascular developmental defect in* Tie2-R848W^fl/fl^;Tie2^Cre+^* mice. (A)** Heatmap of differentially expressed genes in pulmonary vascular developmental defects from *Tie2-R848W^fl/fl^;Tie2^Cre+^* mice versus normal lung tissue from control mice. Each column represents an individual replicate, and each row represents an individual gene. Upregulated genes are shown in red, and downregulated genes are displayed in blue. n=4 mice for the pulmonary vascular developmental defect group, n=3 mice for the control group. Differentially expressed genes were defined as genes with a Benjamini‒Hochberg-adjusted *P*<0.05 and |log_2_FoldChange|>1. **(B)** Volcano plots of gene expression changes in pulmonary vascular developmental defects versus normal lung tissue from control mice. Green dots indicate downregulated genes. Red dots indicate upregulated genes. *P*<0.05 and |log_2_FoldChange|>1. *P* values were determined by the limma package. n=4 mice for the pulmonary vascular developmental defect group, n=3 mice for the control group. **(C)** KEGG pathway classifications of differentially downregulated genes. **(D)** Top 10 GO terms related to biological processes for significantly downregulated genes. **(E)** Top 18 KEGG pathways of differentially downregulated genes. **(F)** Heatmap of genes with the most obvious downregulation and genes related to phenotypic transition of VSMCs. Each column represents an individual replicate, and each row represents an individual gene. Upregulated genes are shown in red, and downregulated genes are displayed in blue. n=4 mice for the pulmonary vascular developmental defect group, n=3 mice for the control group.

To investigate the molecular mechanism of pulmonary vascular malformations with internal hemorrhage in* Tie2-R848W^fl/fl^;Tie2^Cre+^* mice, transcriptome (RNA) sequencing analysis was performed on lesion tissue from mice with pulmonary vascular malformations (W4) compared with normal lung tissue from control mice (Figure 3A). The sequencing results showed that the *Tie2* mutation background induced significant downregulation of 126 genes in pulmonary vascular malformations and significant upregulation of more than 62 genes (Figure 3B). KEGG pathway classification analysis implied that the downregulated genes were highly enriched and concentrated in the circulatory system (11%) and cardiovascular system (12%) (Figure 3C), while no upregulated genes were significantly enriched in vascular development-related patterns (Figure S10). Thus, Gene Ontology (GO) function analysis of these significantly downregulated genes was further performed to investigate the potential cellular phenotype or foundation that might result in vascular malformations. Consistent with the aforementioned KEGG pathway analysis results, the biological process terms associated with the downregulated genes were mainly related to muscle contraction function, including sarcomere composition, muscle contraction, calcium channel, and other pathway terms. These pathways were closely related to the biological function of vascular smooth muscle but not endothelial proliferation or smooth muscle recruitment, as previously understood (Figure 3D). Within the top 20 upregulated pathways in KEGG enrichment analysis, related genes in the Jak-STAT signaling pathway and PI3K-AKT pathway were obviously upregulated (Figure S10), consistent with previously reported *in vitro* results 3, 7, 15.

Within the top 18 downregulated pathways in KEGG enrichment analysis were obviously enriched nodes including dilated cardiomyopathy (DCM), hypertrophic cardiomyopathy (HCM), cardiac muscle contraction, and the calcium signaling pathway. These processes are all involved in the regulation of contractile function and the phenotype of smooth muscle (Figure 3E). According to the KEGG pathway enrichment results, we further screened and analyzed related genes with the most obvious downregulation (the *p* value < 0.05 and the fold change > 2), and found that most genes closely related to smooth muscle contraction function, myoglobulin/troponin, and muscle filament gliding were indeed downregulated (Figure 3F). Changes in vascular smooth muscle contractility are usually accompanied by a phenotypic transition from contractile to synthetic VSMCs. Interestingly, the contractile markers *Acta2* (Actin, aortic smooth muscle, Sma), *Cnn1* (Calponin 1, Cnn1), and *Tagln* (Transgelin, Sm22a) were slightly downregulated, while the synthetic marker *Spp1* (Osteopontin, Opn) was obviously upregulated (Figure 3F).

The above information indicated that dysregulated function and phenotypic transition of vascular smooth muscle might be the pathogenic basis of *Tie2* mutation-related vascular malformations.

### HUVECs carrying *TIE2-R849W* demonstrate the phenotypic transition of human umbilical vein smooth muscle cells (HUVSMCs)

**Figure 4:**
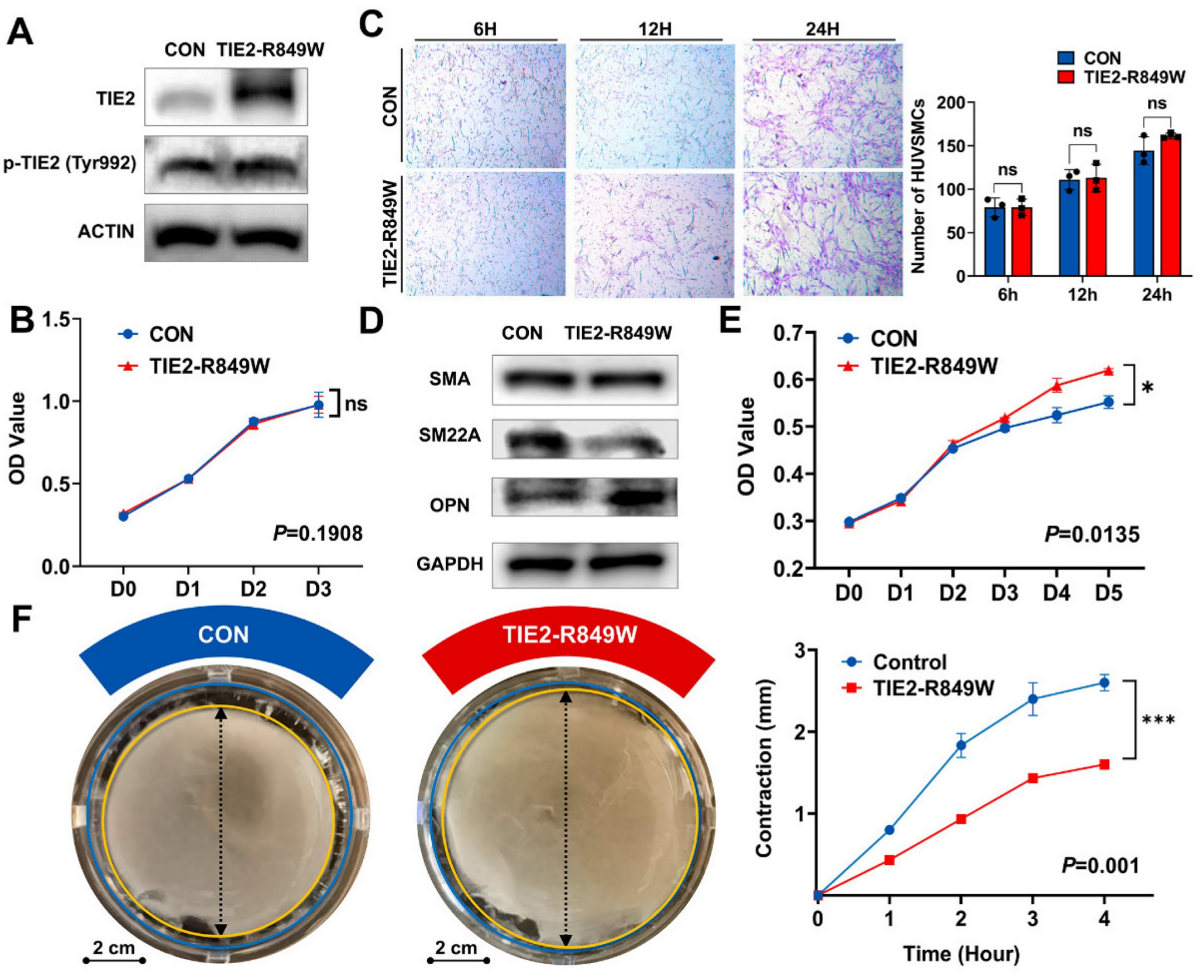
**HUVECs carrying *TIE2-R849W* demonstrated the phenotypic transition of HUVSMCs. (A)** The protein expression level and phosphorylation state of nontransfected HUVECs (CON group) and HUVECs transfected with *TIE2-R849W* (TIE2-R849W group) probed with the indicated antibodies (TIE2, p-TIE2, and ACTIN). **(B)** *TIE2* mutation did not obviously affect the proliferation ability of nontransfected HUVECs (CON) and HUVECs transfected with *TIE2-R849W* (TIE2-R849W). The data are expressed as the mean±SEM. ns, not significant. **(C)** *TIE2* mutation did not obviously affect the recruitment of VSMCs under the HUVEC-HUVSMC coculture conditions (6 h, 12 h and 24 h). Compared with that in the control group, the protein expression of SMA, SM22A and OPN was altered **(D)**; proliferation ability was increased **(E)**; and contractile ability was reduced **(F)** for HUVSMCs that were cultured with conditioned medium from HUVECs transfected with *TIE2-R849W* (TIE2-R849W). Bar=2 cm. The data are expressed as the mean±SEM. *P<0.05, **P<0.001, ***P<0.0001, ns, not significant.

Although the transgenic mouse model confirmed the molecular characteristics of phenotypic transition in vascular smooth muscle due to the *TIE2* mutation, we wanted to further eliminate the potential influence of altered recruitment ability of VSMCs. Therefore, through virus transfection, we used HUVECs to construct the classic *in vitro* model of the* TIE2-R849W* mutation^15^. The overexpression and activated phosphorylation of mutant *TIE2* were verified as previously reported^15^, ^17^ (Figure 4A). *TIE2* mutation did not obviously affect the proliferation of ECs (Figure 4B). Based on the coculture conditions, the number of recruited VSMCs was also not obviously altered (Figure 4C), and there was no significant change in the protein expression level of SMA. However, consistently, the contractile marker SM22A was significantly downregulated, while the synthetic marker OPN was obviously upregulated (Figure 4D). Moreover, under the influence of the *TIE2* mutation, VSMC proliferation was obviously increased (Figure 4E). Another vital representative phenotype of phenotypic transition, the contractile ability of VSMCs, was decreased significantly in the mutant group (Figure 4F). In conclusion, integration of molecular characteristics and cellular phenotypes revealed that the endothelial mutation *TIE2-R849W* induced an obvious phenotypic transition in VSMCs.

### Downregulated ion transmembrane transport and TNFSF10 might be deeply involved in initiating the phenotypic transition process in VSMCs

**Figure 5:**
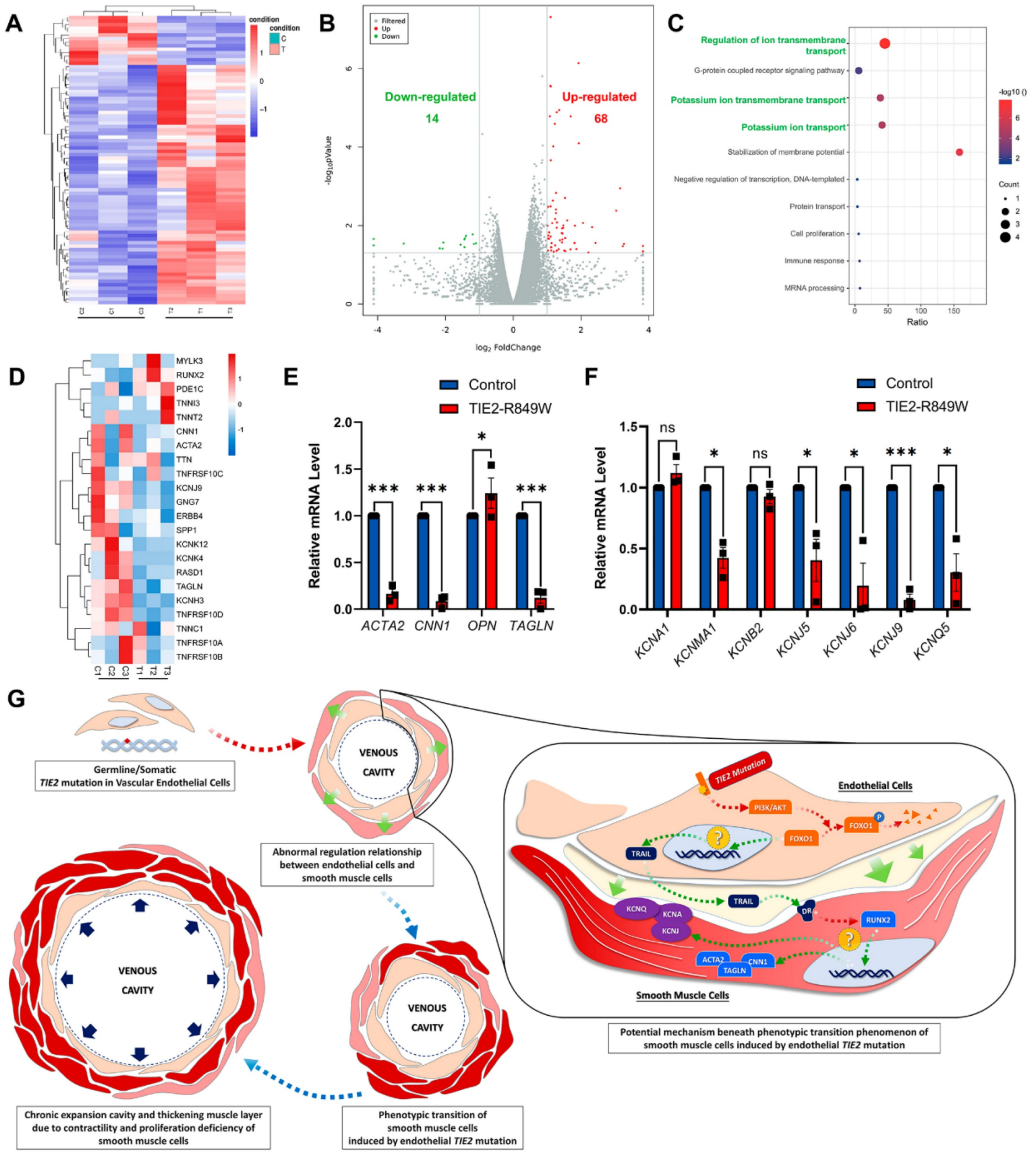
** Downregulated ion transmembrane transport might be deeply involved in initiating the phenotypic transition process in VSMCs. (A)** Heatmap of differentially expressed genes in VSMCs cocultured with conditioned medium from control or mutant HUVECs in advance for 48 hours. Each column represents an individual replicate, and each row represents an individual gene. Upregulated genes are shown in red, and downregulated genes are displayed in blue. Differentially expressed genes were defined as genes with a Benjamini‒Hochberg-adjusted *P*<0.05 and |log_2_FoldChange|>1. **(B)** Volcano plots of gene expression changes in VSMCs cocultured with conditioned medium from control or mutant HUVECs in advance for 48 hours. Green dots indicate downregulated genes. Blue red dots indicate upregulated genes. *P*<0.05 and |log_2_FoldChange|>1. The *P* values were determined by the limma package. **(C)** Top 10 KEGG pathways of differentially downregulated genes. **(D)** Heatmap of genes with the most obvious downregulation and genes related to phenotypic transition of VSMCs. Each column represents an individual replicate, and each row represents an individual gene. Upregulated genes are shown in red, and downregulated genes are displayed in blue. Validation of the mRNA expression pattern of genes related to the phenotypic transition of VSMCs (*ACTA2*, *CNN1*, *OPN* and *TAGLN*) **(E)** and downregulated genes closely related to ion transmembrane transport (*KCNA1*, *KCNMA1*, *KCNB2*, *KCNJ5*, *KCNJ6*, *KCNJ9* and *KCNQ5*) **(F)**. *P<0.05, **P<0.001, ***P<0.0001, ns, not significant. **(G)** Schematic diagrams depicting that germline/somatic* TIE2* mutation in ECs might induce an abnormal regulatory relationship between ECs and VSMCs that is closely associated with the phenotypic transition of VSMCs. Abnormal contractility and proliferation due to increased synthetic VSMCs induce chronic expansion of the cavity and thickening of the muscle layer. The mechanical pressure on the wall and a hypoxic environment might also stimulate the synthesis transition and eventually result in VMs.

An exploratory analysis was performed to identify the underlying molecular mechanism that triggers the phenotypic transition of VSMCs with the *TIE2-R849W* mutation. Transcriptome (RNA) sequencing was performed on VSMCs cocultured with conditioned medium from control or mutant HUVECs in advance for 48 hours (Figure 5A). Unlike in the *in vivo* mouse model, in which most genes were suppressed, 14 genes were significantly downregulated in the* in vitro TIE2* mutation background, whereas 68 genes were upregulated (Figure 5B). Within the top 10 downregulated pathways in KEGG enrichment analysis, genes related to ion transport (regulation of ion transmembrane transport, potassium ion transmembrane transport, and potassium ion transport) were highly affected; these findings were in contrast to the *in vivo* regulation of contractile function (Figure 5C). We further screened and analyzed related genes with the most obvious regulation according to the KEGG pathway enrichment results. On the one hand, the contractile markers *ACTA2*, *CNN1* and *TAGLN* were significantly downregulated at the mRNA level, while the synthetic marker *OPN* was upregulated (Figure 5D and E). On the other hand, most genes closely related to ion transmembrane transport, especially potassium ion transport, were significantly downregulated (Figure 5D and F).

The details of the crosstalk between mutant ECs and VSMCs are still uncertain. Previously, researchers have conducted transcriptomic sequencing analyses on ECs carrying the *TIE2-R849W* mutation (GSE46684)^15^. To exclude the influences of other cell types, consistent molecular changes in ECs under the influence of *TIE2* mutation should be identified *in vivo* and *in vitro*. Therefore, a cross-comparison of these two RNA sequencing datasets was performed, and 25 consistently and significantly altered genes were found (Figure S11A). Subsequently, based on consistent variation trends and phenotypic correlation analysis, tumor necrosis factor superfamily 10 (TNFSF10 TRAIL) was the only significantly differentially expressed gene closely related to the smooth muscle phenotypic transition (significantly downregulated both *in vivo* and *in vitro*)^18^, ^19^ (Figure S11B). Under coculture conditions, the protein expression level of TNFSF10 was downregulated in ECs (Figure S11C). to address the potential regulatory relationship between FOXO1 and TNFSF10 expression, we performed computational prediction of FOXO1 binding motifs using the JASPAR database. The analysis revealed three evolutionarily conserved FOXO1 binding sites within the TNFSF10 promoter region (positions 58-71, 249-261, 365-378 relative to the transcription start site), suggesting a direct transcriptional regulatory mechanism. To experimentally validate this prediction, we employed a selective FOXO1 inhibitor (AS1842856) that suppresses FOXO1 transcriptional activity by stabilizing its cytoplasmic retention through competitively binding to FOXO1. Western blot analysis confirmed effective reduction of both total FOXO1 protein levels and its phosphorylated form (p-FOXO1 Ser256) in the inhibitor-treated group. Subsequent TNFSF10 protein quantification revealed that FOXO1 inhibition recapitulated the attenuated TNFSF10 expression observed in the mutant group (Figure S12). However, the underlying mechanism of the inhibition of TNFSF10 and smooth muscle phenotypic transition remains to be further studied in the context of *TIE2* mutations (Figure 5G).

In conclusion, germline/somatic* TIE2* mutations in ECs might induce an abnormal regulatory relationship between ECs and VSMCs that is closely associated with the phenotypic transition of VSMCs. Abnormal contractility and proliferation due to increased numbers of synthetic VSMCs induce chronic expansion of the cavity and thickening of the muscle layer. The mechanical pressure on the wall and a hypoxic environment might stimulate the synthesis transition and eventually result in VM formation (Figure 5G).

## Discussion

*TIE2-R849W* is a classical mutation associated with VMs, and there is sufficient genetic evidence for its inheritance pattern in VMCM. Thus, it is of substantial clinical and scientific value to carry out further basic research. In this study, by designing two mutation knock-in strategies, we simulated two different mutation mechanisms with germline and somatic patterns. According to the spatiotemporal distribution of *Tie2*, all tissues or organs closely related to the vasculature underwent detailed phenotypic screening and histological identification. Finally, the phenotypic characteristics of pulmonary vascular malformations induced by *Tie2* mutation were clarified. Relatively abundant expression of *Tie2*, an important marker of pulmonary vascular endothelial cells that was induced by the mutation background, was the internal cause of vascular malformation. A previous single-cell sequencing analysis^20^ suggested that Tie2 and Apln are highly expressed in general capillary cells, which form the main cellular component of pulmonary capillaries. With the development of the lungs (E15.5 to P1), the expression level of Tie2 gradually increases, and the expression tends to be stable after birth^21^. Another study on bronchial dysplasia-related vascular abnormalities has confirmed^20^, ^22^ that TIE2 is expressed only in vascular ECs, not in the alveolar or airway epithelium. The number of vascular clusters (density of the microvasculature) surrounding the alveolar lumen is positively correlated with the expression and function of TIE2^22^. Therefore, pulmonary vascular development could be significantly affected by Tie2. Based on our previous clinical observational studies on patients with sporadic and familial hereditary venous malformations carrying *TIE2* mutations (results not presented), combined with previous literature reports, no clinical phenotypes of pulmonary venous malformations have been identified in patients. In addition, interestingly, similar to the pulmonary vascular malformations in this study, abnormal vascular development has been previously identified in patients and mouse models related to hereditary telangiectasia (HHT)^23^. The generation and development of HHT are also closely related to the TIE2 signaling pathway^24^. Therefore, studying the effect of *TIE2* mutation on vascular development via the pulmonary vascular malformation phenotype is a reliable and representative strategy.

In addition to pulmonary vascular malformation models, the retinal vascular model is currently a mainstream *in vivo* model for observing the early angiogenesis process. Retinal vascularization has been observed and analyzed in another published *Pik3ca^H1047R^* transgenic mouse model associated with VMs^9^, ^10^. Similar to the *Pik3ca* mutation model, *Tie2*-mutant mice also showed significantly reduced retinal vascular outgrowth. In contrast, *Pik3ca-*mutant mice exhibit less VSMC coverage than normal mice, but the coverage of VSMCs in *Tie2-R848W^fl/fl^* mice was significantly higher than that in the control group. Subsequently, based on an *in vitro* model, the potential enhanced recruitment process of VSMCs was excluded. Combined with the histological features of the pulmonary phenotype, the findings comprehensively demonstrated that under the influence of *TIE2* mutation, the proliferation ability of VSMCs was upregulated (synthetic transition). Therefore, although the clinical manifestations of VMs are similar, the pathological types, pathogenic mechanisms, and initiating factors are entirely different between lesions carrying *PIK3CA* mutations and those with *TIE2* mutations. These differences might partly explain the various responses of VMs to mTOR inhibitors (rapamycin/sirolimus). VMs of the extremities and lymphatic malformations with higher *PIK3CA* mutation rates are more responsive to rapamycin treatment, while most VMs with *TIE2* mutations are not as sensitive^25^.

According to physiological functions and cellular phenotypes, VSMCs can be divided into the contractile type and synthetic type, and these two phenotypes are interchangeable under diverse physiopathological influences^26^. Contractile VSMCs are highly differentiated and unable to proliferate. Contractile VSMCs commonly exhibit enhanced contractile ability. The molecular markers of the contractile type mainly include α-SMA (ACTA2), calponin (CNN1), SM22-A (TAGLN) and SM-MHC. These molecules are deeply involved in the composition or function of the cytoskeleton and actin/myosin. In contrast, synthetic VSMCs are dedifferentiated and exhibit proliferation ability. Synthetic VSMCs participate in repairing injured vessels and synthesizing matrix components, but their contractile function is obviously weaker than that of contractile VSMCs. Molecular markers of synthetic VSMCs include OPN (SPP1), MGP and other molecules. OPN is the most abundant and important molecule in synthetic VSMCs. The upregulation of OPN in the mouse model largely confirmed the abnormal phenotypic transition of VSMCs. Abnormal smooth muscle phenotypic transition is often closely related to atherosclerosis, aortic dissection/aortic aneurysm, stroke, tumor angiogenesis and other pathological vascular processes^26^-^28^. Previous studies on persistent lumen dilatation in VMs have focused on the scarcity or uneven distribution of VSMCs due to interference with the recruitment of VSMCs. However, this study implemented a novel approach by focusing on functional alterations in VSMCs. Analyses of pulmonary/retinal vascular malformations and *in vitro* model experiments comprehensively described the phenotypic transition of VSMCs under the influence of *TIE2* mutation. In other words, the aberrant regulation of function and phenotype in VMs was described.

To date, only one high-throughput sequencing study on VMs has used HUVECs with gene mutations^15^. This genetic background is relatively well known, which aids in exploration of the effects of mutations on EC. However, HUVECs cannot reflect the molecular interactions among various cells in a complex cellular environment. Another study has directly used VM lesions for analysis^29^. The relevant results can be used to analyze the overall molecular characteristics of lesions and reflect the complex regulatory status of ECs or EC-VSMCs. However, the unknown genetic background makes it difficult to reach general conclusions. Therefore, aiming to identify the mechanism of phenotypic transition induced by *TIE2* mutations, this study combined the advantages of these two types of high-throughput sequencing analysis to cross-analyze data from *in vivo* and *in vitro* models. The analysis identified TNFSF10 as the only significantly differentially expressed gene closely associated with phenotypic transition. Previous studies^18^, ^30^, ^31^ have suggested that this molecule is deeply involved in the regulation of calcium channels, calcium storage and the contractile function of VSMCs. Specifically, TNFSF10 can utilize calcium channels to dynamically regulate calcium storage. As typical ion transmembrane transport channels, large-conductance voltage and Ca^2+^-dependent K^+^ channels (BK channels) have been shown to regulate different VSMC phenotypes and functional statuses by forming complexes with calcium channels^32^. A previous study^33^ has suggested that downregulation of CaV3 and BK channel-related subunits significantly decreases the contractile ability of VSMCs. Our transcriptomic analysis revealed significant down-regulation of multiple calcium/potassium channel subunits (including CaV3 and BK channel-associated components) in VSMCs exposed to mutant endothelial cells (ECs). This finding aligns with prior reports demonstrating that reduced expression of CaV3 and BK channel subunits compromises VSMC contractility by disrupting calcium signaling dynamics. We hypothesize that mutant TIE2 may induce intracellular calcium dysregulation and membrane potential instability through this pathway, potentially contributing to the observed vascular abnormalities. Supporting this hypothesis, our preliminary investigations detected aberrant calcium distribution patterns in mutant ECs (calcium flux data not presented here).

Since TNFSF10 transcription is clearly regulated by the FOXO-related pathway^34^, *TIE2* mutation can trigger the phosphorylation and degradation of FOXO1^13^, ^15^, which may be the direct explanation for the downregulation of *TNFSF10*. TNFSF10 (TNF-related apoptosis-inducing ligand, also known as TRAIL), a member of the tumor necrosis factor (TNF) superfamily, functions as an extracellularly secreted cytokine with potent apoptotic and differentiation-inducing capabilities in both autocrine and paracrine contexts 35, 36. The reduction of TNFSF10 secretion has been shown to enhance cellular anti-apoptotic and proliferative capacities, contributing to pathological cellular behaviors. In *vivo* studies and clinical observations have further demonstrated that TNFSF10 plays a crucial protective role during vascular development 37. Specifically, transgenic mouse models with targeted *Tnfsf10* gene knockout exhibit retinal vascular malformations in neonates, phenocopying the vascular abnormalities observed in *Tie2* mutant mouse models 18. Notably, both models display a characteristic increase in perivascular smooth muscle cell accumulation, suggesting a potential mechanistic link between TNFSF10 deficiency and vascular remodeling. In our study, we have extended these findings by employing an *in vitr*o model system to investigate the molecular mechanisms underlying TNFSF10 regulation in the context of *TIE2* mutation. At the protein level, we have demonstrated that *TIE2* mutation leads to significant downregulation of TNFSF10 expression. Furthermore, we have provided preliminary evidence suggesting that FOXO1, a downstream effector in this pathway, may mediate the observed regulatory effects (Figure S12). These findings not only corroborate previous reports but also establish a novel mechanistic connection between TIE2 signaling and TNFSF10-mediated vascular homeostasis. To date, the functional regulation of VSMCs in VMs has not been studied deeply. Therefore, to further verify the direct regulatory network of and effects of *TIE2* mutations on TNFSF10, it will be necessary to comprehensively elucidate the roles and mechanisms of calcium/potassium channels in smooth muscle changes. This will enable exploration of the possible mechanisms of TNFSF10 on the phenotypic transition of VSMCs. Research in this field will contribute greatly to explaining and exploring the novel pathogenic mechanism of VMs.

## Conclusions

Based on the novel transgenic mouse model of *TIE2* mutation associated with VMs, pulmonary vascular malformations and postnatal retinal vascular malformations under the influence of mutation were discovered and identified. According to results of high-throughput transcriptome sequencing analysis of pulmonary lesions, retinal vascular development analysis and cell model, germline/somatic *TIE2* mutation in ECs might induce an abnormal EC-VSMC regulatory relationship strongly associated with the VSMC phenotypic transition, which is different from other types of VMs. Downregulated ion transmembrane transport and TNFSF10 might be deeply involved in initiating the phenotypic transition process in VSMCs. Weakened contractility and abnormal proliferation induce chronic cavity expansion and thickening of the muscle layer, which may finally result in VMs.

## Supplementary Material

Supplementary figures and tables.
