# Supplementary material for: Endothelial TIE2 Mutation Induced Contraction Deficiency of Vascular Smooth Muscle Cells via Phenotypic Transition Regulation in Venous Malformations
Source: Int J Med Sci. 2025 May 7;22(10):2518–32. doi: 10.7150/ijms.102700 (PMC12080580; doi:10.7150/ijms.102700)
Supplement: Supplementary file 1 — Supplementary figures and tables. [file ijmsv22p2518s1.pdf]

Supplementary Material

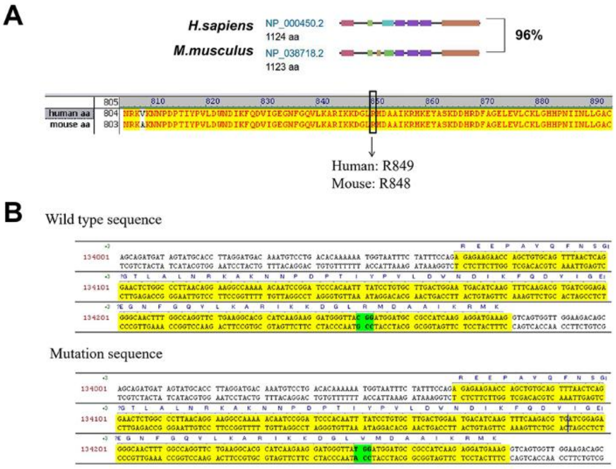

Figure S1 Homologous comparison of human/mouse *TIE2* gene (A) and identification of *R849W* mutation site. (B).

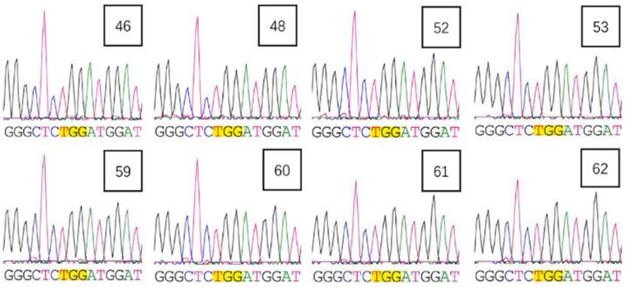

Figure S2 F1 generation mice accepted Sanger Sequencing to confirm target genetic variant (*CGG>TGG*).

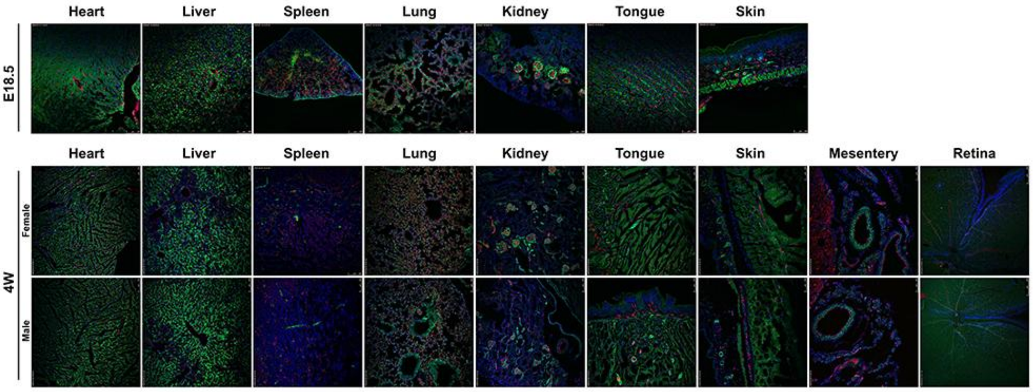

Figure S3 Efficiency and expression of Cre enzyme in *Tie2-Cre*$^{+}$ transgenic mouse model during the embryonic period (embryo 18.5 days) and the immature period (4 weeks).

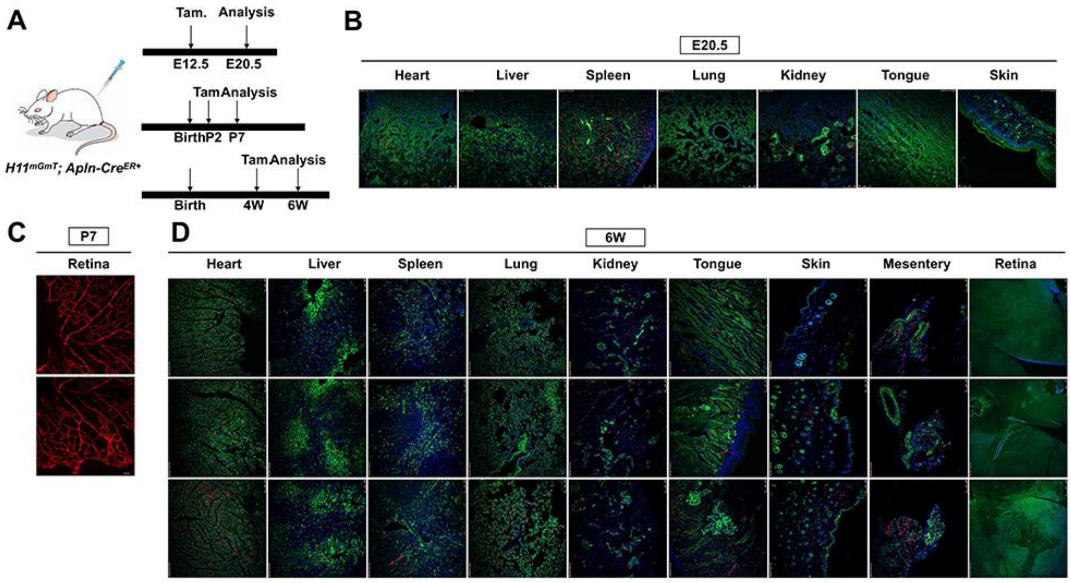

Figure S4 Efficiency and expression of Cre enzyme in *Apln-Cre*^{ER} transgenic mouse model. (A) Schematic of the tamoxifen (Tam.) administration and analysis designed. Efficiency and expression of Cre enzyme in *Apln-Cre*^{ER} transgenic mouse model during embryo 18.5 days (B), postnatal 7 days (C) and 6 weeks (D).

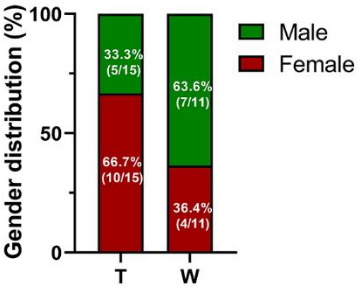

Figure S5 Female and male proportion of homozygous mutant mice (*Tie2-R848W$^{fl/fl}$;Tie2^{Cre+}* mice) compared with control group.

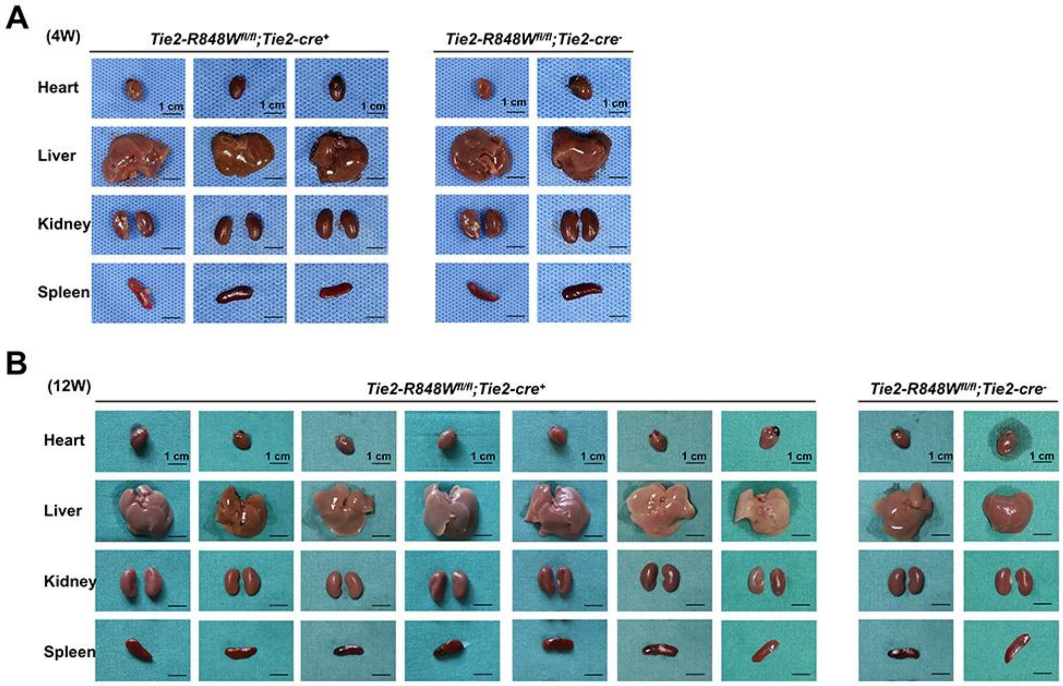

Figure S6 Anatomy and observation of the internal organs (lung, heart, liver, kidney, spleen and other organs that are histologically rich in vascular tissue) at 4 weeks (A) and 12 weeks (B).

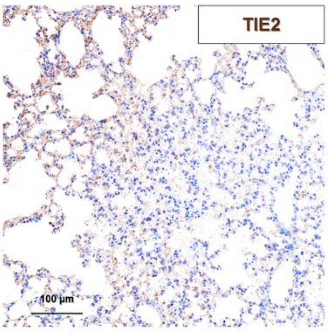

Figure S7 The positive immunohistochemical expression of TIE2 was confirmed in *Tie2-R848W*$^{fl/fl}$*;Tie2*$^{Cre+}$ mice.

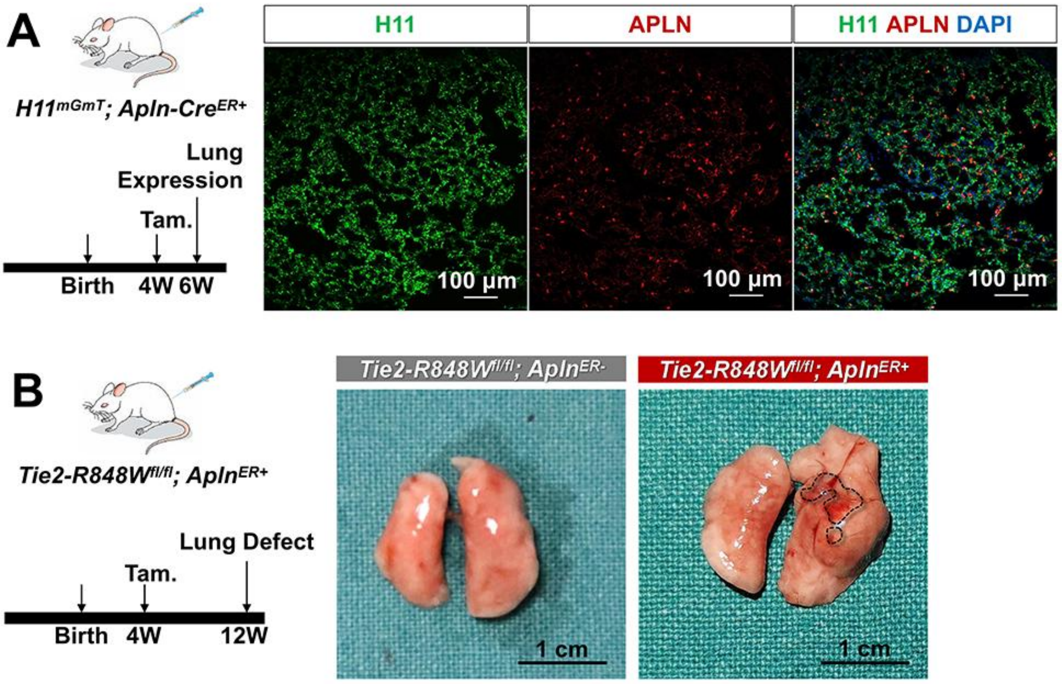

Figure S8 *Tie2-R848W*$^{fl/fl}$*;Apln*$^{ER+}$ mice developed pulmonary vascular malformations with internal hemorrhage. (A) As schematic of the tamoxifen (Tam.) administration and analysis designed (4 weeks), efficiency and expression of Cre enzyme in *Tie2-R848W*$^{fl/fl}$*;Apln*$^{ER+}$ mouse model during the immature period (6 weeks) were analyzed. Bar=100 μm. (B) As schematic of the tamoxifen (Tam.) administration and analysis designed (4 weeks), similar but slighter pulmonary vascular malformations with internal hemorrhage (surrounded by black dotted line) were also identified in *Tie2-R848W*$^{fl/fl}$*;Apln*$^{ER+}$ *mice* at 12 weeks. Bar=1 cm.

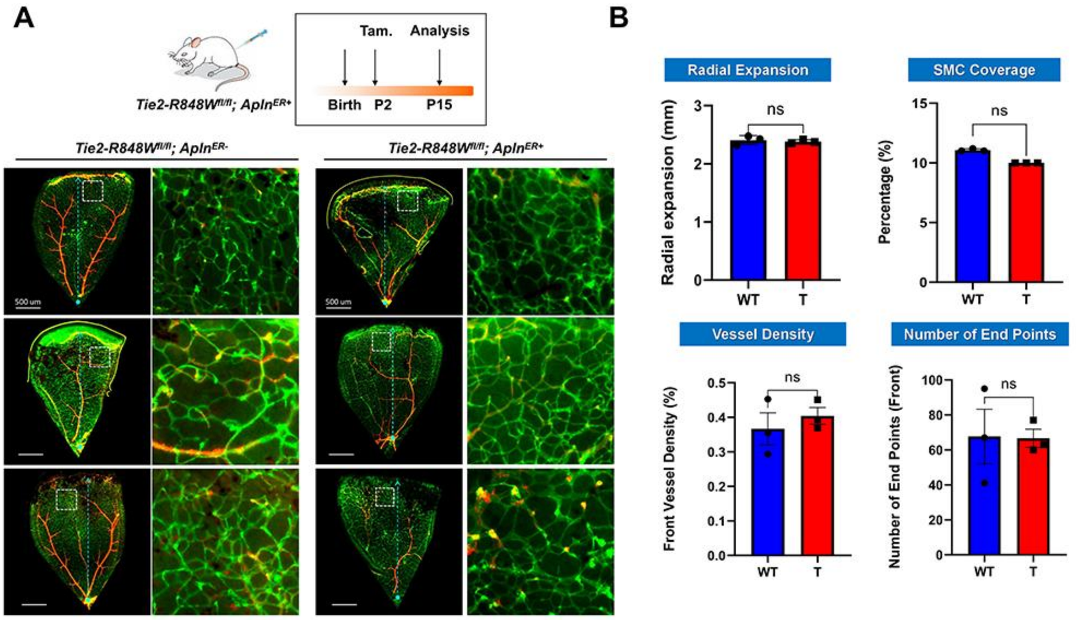

Figure S9 Endothelial cell specific *Tie2-R848W* induced postnatal retinal vascular malformations in P15 stage. (A) Schematic of the tamoxifen (Tam.) administration and analysis designed in the *Tie2-R848W*$^{fl/fl}$;*Apln*$^{ER+}$ mice for postnatal retinal vascular analysis (P15). Immunofluorescence colocalization analysis of postnatal retinal vascular (P15) for CD31(green) and α-SMA (orange) in the *Tie2-R848W*$^{fl/fl}$;*Apln*$^{ER+}$ mice were performed. No obvious difference was observed, implying that *Tie2-R848W* might significantly influence the early angiogenesis process but not vascular reconstruction. Blue dashed arrow, the radical expansion of retinal vasculature from the outer front edge of the vascular network to the center of the optic axis. Red dashed arrow, distance between retinal vasculature margin and retinal margin (reduced retinal vascular outgrowth). Square region surrounded by white dotted line, typical retinal vascular growth front region. Bar=100 μm. (B) Compared with the control group, there was no significant difference from the aspects of either radical expansion, SMC coverage rate, vascular density of the developmental

frontier area or the number of neovascular buds (the number of end points).

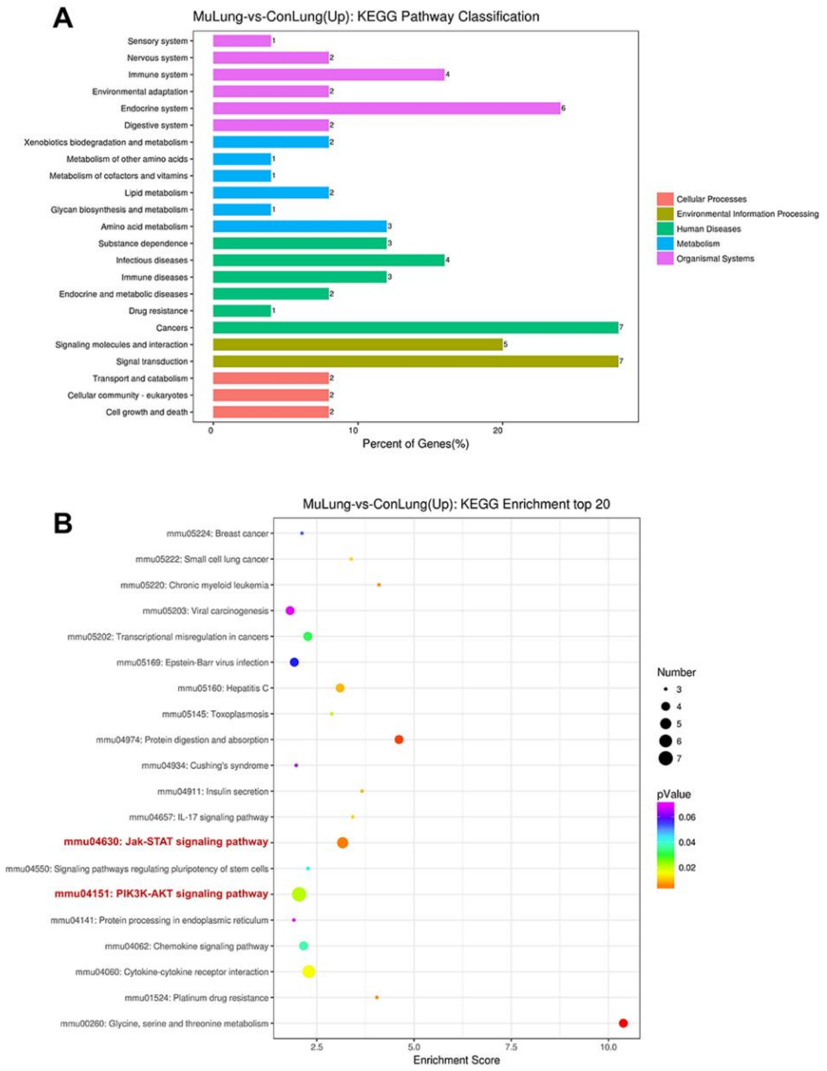

Figure S10 Transcriptomic bioinformatics analyses for upregulated genes in pulmonary vascular malformations from *Tie2-R848W*$^{fl/fl}$;*Tie2*$^{Cre+}$ mice versus normal lung tissue from control mice. (A) The Kyoto Encyclopedia Genes and Genomes (KEGG) pathway classification of differentially upregulated genes. (B) The top 20 KEGG pathways of differentially upregulated genes.

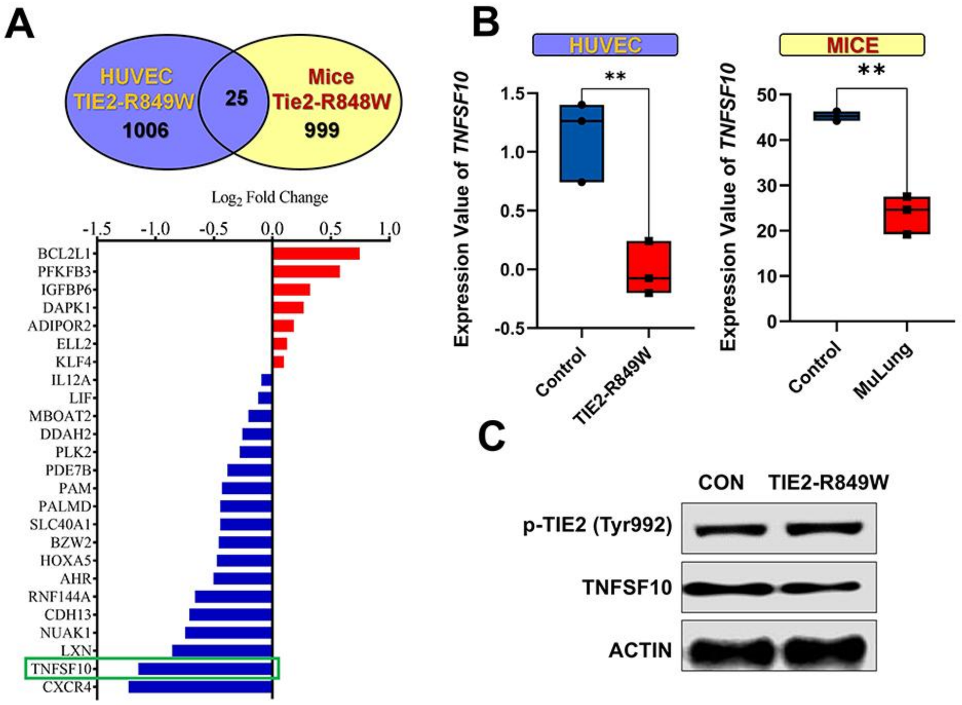

Figure S11 TNFSF10 might perform a vital crosstalk role between mutant ECs and VSMCs for dysregulated function and phenotypic transition of VSMCs. (A) Venn diagram indicating 25 consistent differential expression genes shared by HUVECs carrying the *TIE2-R849W* mutation (GSE46684) and pulmonary vascular malformations from *Tie2-R848W*$^{fl/fl}$*;Tie2*$^{Cre+}$ mice. Upregulated genes are shown in red, and downregulated genes are displayed in blue. (B) Significantly downregulated mRNA expression of *TNFSF10* in *in vivo* and *in vitro* transcriptomic sequencing array. ***P*<0.001. (C) The related protein expression level and phosphorylation state alteration of nontransfected HUVECs (CON group) and HUVECs transfected *TIE2-R849W* (TIE2-R849W group) probed with indicated antibodies (p-TIE2, TNFSF10, ACTIN).

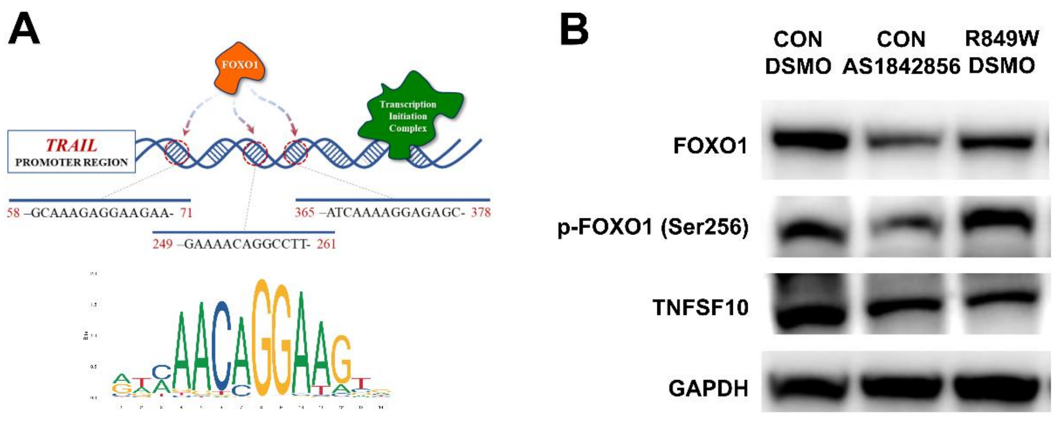

Figure S12 Mutant TIE2 may affect the abnormal expression of TNFSF10 through FOXO1 pathway. (A) The online software JASPAR predicted the binding motifs of FOXO1. The analysis revealed three evolutionarily conserved FOXO1 binding sites within the *TNFSF10* promoter region (positions 58-71, 249-261, 365-378 relative to the transcription start site). (B) The related protein expression level and phosphorylation state alteration of nontransfected HUVECs (CON DSMO group), HUVECs treated with FOXO1 inhibitor (CON AS1842856 group) and HUVECs transfected *TIE2-R849W* (R849W DSMO group) probed with indicated antibodies (FOXO1, p-FOXO1, TNFSF10, GAPDH).

Table S1. Primer design for qPCR.

Gene	Primer (5'to3')
ACTA2	
Forward	AAAAGACAGCTACGTGGGTGA
Reverse	GCCATGTTCTATCGGGTACTTC
CNN1	
Forward	CTGTCAGCCGAGGTTAAGAAC
Reverse	GAGGCCGTCCATGAAGTTGTT
TAGLN	
Forward	AGTGCAGTCCAAAATCGAGAAG
Reverse	CTTGCTCAGAATCACGCCAT
OPN	
Forward	CTCCATTGACTCGAACGACTC
Reverse	CAGGTCTGCGAAACTTCTTAGAT
KCNA1	
Forward	CATCGTGGAAACGCTGTGTAT
Reverse	AACCCTTACCAAGCGGATGAC
KCNMA1	
Forward	GGCAGCAGTCTTAGAATGAGTAG
Reverse	AAAGCCCACCACATGCGTT
KCNB2	
Forward	GGGCCTCAACCACGAAGTC

Reverse	TCCTGGATGCCGATCAAAGAA
KCNJ5	
Forward	GCTGGCGATTCTAGGAATGC
Reverse	TCTGTGGCAATGGGGACATAA
KCNJ6	
Forward	GACAGAATCCATGACTAACGTCC
Reverse	CTGGCCTGCTTAGGCAACTTT
KCNJ9	
Forward	AACGTGCGCGAGACATACC
Reverse	AGGACGAAGAACAACAGGCTG
KCNQ5	
Forward	AAGCCGCTCTCTTACACGAG
Reverse	AGAAAAACGAAAGCGTGGTAGAT
GAPDH	
Forward	GGAGCGAGATCCCTCCAAAAT
Reverse	GGCTGTTGTCATACTTCTCATGG
